# Traditional wisdom through scientific evidence: a review of *Marma* manipulation in *Amsa sandhimokśa* (shoulder joint dislocation) with a special focus on the Milch method

**DOI:** 10.3389/fmed.2026.1778769

**Published:** 2026-03-20

**Authors:** Sruthi Chenganakattil, Soumya Mohan, Shyam Vinayan

**Affiliations:** Department of Shalya Tantra, Amrita School of Ayurveda, Kollam, Kerala, India

**Keywords:** *Amsa sandhimokśa*, Ayurveda, *Marma* manipulation, Milch method, shoulder dislocation

## Abstract

**Introduction:**

Shoulder dislocations represent 50 percent of all major joint dislocations, with anterior dislocation being most common accounting for up to 97% of all shoulder dislocations. The shoulder is inherently unstable because the shallow glenoid fossa articulates with only a small portion of the humeral head. Various techniques are available for reduction, with Kocher’s being widely practiced. However, both the Kocher and Hippocratic techniques are linked to complications like axillary nerve injury and humeral fractures. Traditional *Vaidyas* utilize several techniques, notably one similar to the rarely used Milch technique, which has been in practice for over 70 years. Studies show that the Milch technique demonstrates a higher success rate and a shorter time to achieve reduction without sedation compared with the Stimson technique. This review paper aims to investigate the integration of traditional healing practices, specifically *Marma Chikitsa* manipulation, with contemporary scientific principles.

**Methodology:**

The relevant papers regarding shoulder joint dislocation were reviewed from scholarly articles.

The concept of *Amsa sandhimokśa* was reviewed in various Ayurvedic treatises.

**Results:**

The *Marma* manipulation technique closely resembles the Milch technique in its biomechanical principle of utilizing leverage and controlled positioning rather than forceful traction, thereby facilitating effective correction with minimal pain and reduced need for sedation.

**Discussion:**

The various methods for addressing anterior shoulder joint dislocation should be carefully considered.

**Conclusion:**

It is reported to be relatively atraumatic and painless, with a success rate comparable to more established methods.

## 1 Introduction

*Marma Chikitsa* Methods are age-old techniques rooted in the rich martial arts traditions of South India. These methods focus on the vital points of the body, known as *“Marma,”* and are designed to enhance physical performance, healing, and overall wellbeing. Today, they are meticulously preserved and practiced by selected families who have inherited this ancient knowledge through generations, ensuring that these valuable techniques remain alive and relevant in contemporary time (1). It is crucial that we take steps to systematically gather and document this information for the preservation and upgradation of Ayurvedic practice. Shoulder dislocation accounts for about 50 percent of all major dislocations, and in 97 percent of the cases will be an anterior dislocation. Shoulder dislocations are common because the shallow glenoid cavity only articulates with a small portion of the humeral head. Many methods are available for reduction among which Kocher’s is widely practiced, even though axillary nerve injury, humeral shaft fractures are seen as major complications (2). In traditional *Marma Chikitsa*, many methods are employed for shoulder reduction, one among which is similar to that of Milch method and has been in practice for over 70 years. So, this paper is to introduce a manipulation technique that is commonly practiced in *Marma Chikitsa* practices.

### 1.1 Ayurvedic concept in the treatment of *Sandhimoksha* (dislocation)

(a) Samanya chikitsa

*Acharya Susrutha* explained the treatment of *Sandhimoksha* in four phases called *Aanchanam* (pulling or traction), *Peetanam* (pressing or compression), *Samkshepam* (approximation), and *Bandhanam* (bandaging):

आञ्छ्नैः पीडनैश्चैव सङ्क्षेपैर्बन्धनैस्तथा ||१८||
सन्धीञ्छरीरे सर्वांस्तु चलानप्यचलानपि |
एतैस्तु स्थापनोपायैः स्थापयेन्मतिमान् भिषक् ||१९||  (suśruta samhitâ cikitsâ sthâna 3/१९-१८)
Translation: By *Aanchana*, *Peetana*, *Samkshepa* and *Bandhana*, by these methods a wise physician can rearrange both movable and immovable joints to their normal positions.

(b) Visesha chikitsa

In shoulder joint dislocation, reduction is achieved by using a *Musala* as a lever to gently elevate and reposition the humeral head into the glenoid cavity. Once proper alignment is restored, an experienced physician applies a *Swasthika bandha* to stabilize the joint, maintain reduction, and support healing (3).

मुसलेनोत्क्षिपेत् कक्षामंससन्धौ विसंहते |
स्थानस्थितं च बध्नीयात् स्वस्तिकेन विचक्षणः ||३१||  (suśruta samhitâ cikitsâ sthâna 3/३१)
Translation: When the shoulder joint has become dislocated, one should use a *Musala* (heavy wooden or metal stick) as a lever to elevate the axilla and the experienced physician should apply a *Swasthika bandha* to stabilize the joint.

(c) Special precautions while managing joint dislocation

When managing a dislocation, it’s important to avoid excessive movement of the joint. Moving it too much can worsen the injury and damage surrounding tissues, nerves, or blood vessels. Careful handling is essential for a safer recovery.

उत्पिष्टमथ विश्लिष्टं सन्धिं वैद्यो न घट्टयेत् |
तस्य शीतान् परीषेकान् प्रदेहांश्चावचारयेत् ||२०||  (suśruta samhitâ cikitsâ sthâna 3/२०)
Translation: If a joint is sprained or dislocated, the physician should not manipulate or force it. Instead, the physician should apply cold therapies like *pradeha* (anointing with cold potency drugs).

According to Acharya Suśruta, in cases of *utpiṣṭa* (fracture-dislocation) and *viśliṣṭa* (subluxation), excessive traction or manipulation should be avoided. In such situations, the application of cold is advised prior to performing the reduction procedure.

### 1.2 Modern techniques for the shoulder reduction

(a) Kocher’s method

The affected arm is first flexed at the elbow and adducted close to the body. The forearm is then externally rotated, followed by elevation of the upper arm in the sagittal plane to the maximum possible extent, and finally completed with internal rotation. This is considered a method that can be administered even without anesthesia (4).

(b) Hippocratic method

With the patient lying supine, the practitioner holds the affected forearm and hand to stabilize the limb and places the heel of the foot in the axilla of the involved shoulder, using it as a controlled fulcrum. While maintaining this fulcrum, gentle and progressive adduction of the arm is performed. This maneuver applies a steady levering force across the shoulder joint, facilitating realignment of the humeral head and thereby initiating reduction of the dislocation (5).

(c) Stimson’s method

The patient is positioned prone on the examining table, with the affected arm allowed to hang freely over the edge. Sustained downward traction is then applied to the limb for approximately 10–20 min, either manually by the practitioner or by suspending weights from the patient’s wrist. This continuous traction facilitates gradual muscle relaxation. Once adequate relaxation is achieved, the weights are released, permitting the humeral head to descend and realign spontaneously into the glenoid fossa, thereby achieving reduction of the glenohumeral joint (5).

(d) Milch method

The patient is placed in a supine position with the shoulders slightly elevated above the pelvis. The practitioner holds the wrist, gently raises the arm overhead, and externally rotates it to approximately 90°. Once this position is achieved, steady pressure is applied to direct the humeral head superiorly and laterally to facilitate reduction (5).

### 1.3 Traditional *Marma* manipulation*-* which is similar to the Milch method

(a) Method of reduction

- Position: supine position with the shoulder close to the edge of the table.
- Abduct the affected arm till the resistance is felt, relax for some time and abduct again till the hand reaches over the head.
- With mild traction, externally rotate the arm.
- Simultaneously push the humoral head with the thumb into the glenoid cavity.
- Bring back the hand with adduction by placing the physician’s hand over the humeral head.
- If the manipulation is difficult due to increased muscle spasm, cold application can be done over the affected shoulder for 5–10 min and then reduction can be tried again.

(b) Differences between Milch and traditional *Marma* manipulation

- In *Marma* manipulation, external rotation is performed with mild traction once the arm is positioned above the head, whereas in the Milch technique, external rotation is carried out in conjunction with abduction.

### 1.4 Principle behind the reduction method

The methods commonly used to reduce a shoulder dislocation are based on two principles: traction and leverage. Traction can increase muscle spasms, requiring more force, which often leads to greater difficulty and pain, making it less likely to succeed. Techniques such as Kocher’s, Hippocratic, and Stimson’s are based on the principle of traction, while the Milch method relies on the principle of leverage. In this method, the positioning of the humerus is established such that the resultant force exerted by the perihumeral muscles aligns parallel to the shaft of the humerus. This alignment effectively minimizes the required muscle force, thereby facilitating a more efficient reduction process when compared to other methods.

## 2 Discussion

### 2.1 Similarities between Milch method and traditional *Marma* manipulation

- The procedure of both the methods is more or less similar. During the *Marma* manipulation mild traction is applied during the abduction of the arm which can be considered as the modification of Milch method.
- After the reduction or even after the reduction doesn’t occur after the step of pushing humeral head to glenoid cavity, the arm will be adducted in such a way that the forearm rest over the chest. In some cases, it is observed that the reduction takes place during this step.
- While performing the reduction (step of adduction) the affected hand will be held over the wrist and the elbow joint which is considered as the site of *manibandha* and *koorpara marmas*. The pressure applications over these regions may facilitate the reduction by reducing the pain perception. According to the Gate Control Theory of pain proposed by Ronald Melzack and Patrick Wall, axons of low-threshold mechanoreceptors (A-beta fibers) and nociceptors (A-delta and C fibers) converge on neurons in the substantia gelatinosa of the dorsal horn. Activation of fast-conducting, myelinated A-beta fibers stimulates inhibitory interneurons that suppress transmission of nociceptive signals to second-order neurons, thereby “closing the gate” and reducing pain perception. As opined by Dr. Bishnupriya Mohanty et al., based on Gate Control Theory, non-noxious stimuli applied to *Marma* points may inhibit the transmission of pain signals to the brain, providing relief from pain (6).

### 2.2 Effectiveness of Milch over other commonly used techniques

- The Milch technique demonstrated advantages over the Stimson technique, achieving successful shoulder reduction without sedation in a greater proportion of patients and within a significantly shorter time frame. These findings suggest that the Milch technique is not only more effective but also more efficient, potentially reducing patient discomfort, minimizing procedural delays, and decreasing the need for pharmacologic sedation in the emergency setting (7).
- In a meta-analysis evaluating eight different techniques for the reduction of anterior shoulder dislocation, the FARES method and scapular manipulation emerged as the most effective approaches in terms of overall success. A noteworthy observation from this analysis is that the initial step of the FARES technique closely resembles the first step of *Marma* manipulation, indicating a procedural similarity that may contribute to its effectiveness.
- Although pain outcomes assessed using the visual analogue scale (VAS) are an important comparative parameter, such an analysis could not be undertaken for the modified Milch technique due to insufficiently reported data in the available literature. Despite this limitation, the Milch method demonstrated a high success rate, with an efficacy of 92.17% (8).
- Regauer et al. noted that the Hippocratic method for anterior shoulder reduction can cause iatrogenic complications, especially neurogenic injury. Traction may bend the neurovascular bundle over the dislocated humeral head, while the operator’s heel can compress vessels against the inferior border of the pectoralis minor, leading to vascular stress or direct injury (9).
- As per Eyal et al., when compared with the Stimson technique, the Milch technique demonstrated a higher success rate and a shorter time to achieve reduction when performed without the use of sedation. This suggests that the Milch method may be more efficient (10).

### 2.3 Effectiveness of cold application before manipulation

- Ayurveda emphasizes the importance of *śīta pariṣeka* before the joint manipulations, it is mainly indicated while dealing with the joint dislocations.
- Cold therapy numbs the area and prevents the muscles from locking up (spasms), making the bone slide back in a much easier way (11).

### 2.4 Effect of minimal traction applied during manipulation

- In the current clinical scenario, greater emphasis is placed on reduction procedures that require minimal traction, as excessive or prolonged traction can increase muscle rigidity. This heightened muscle resistance may, in turn, hinder relaxation around the joint and complicate the reduction process, thereby reducing procedural ease and effectiveness (12).

## 3 Conclusion

- Milch method is reported to be relatively atraumatic and painless, with a success rate comparable to more established methods.
- If muscle spasms prevent the reduction from being performed, śīta pariṣeka must be conducted and later reduction should be performed.
- This method is highly beneficial for both patients and doctors, and it can be effectively performed without the need for an assistant.
- Orthopedics is often perceived as a field for those with a strong physique. However, techniques like this which require less traction can be employed by everyone.
- The method can be considered cost-effective as it uses minimal resources in the form of supportive staff and other medical equipments.

## 4 Recommendations

- This technique deserves greater emphasis in educational settings and practical applications as it is significantly less painful than other reduction methods and less time is required for reduction.
- Enhancing awareness and understanding of this approach could encourage more practitioners to incorporate it into their routine practice.

## Data Availability

The original contributions presented in this study are included in this article/supplementary material, further inquiries can be directed to the corresponding author.
